# The Broken Heart: The Role of Life Events in Takotsubo Syndrome

**DOI:** 10.3390/jcm10214940

**Published:** 2021-10-26

**Authors:** Maria Casagrande, Giuseppe Forte, Francesca Favieri, Francesca Agostini, Jasmine Giovannoli, Luca Arcari, Ilaria Passaseo, Raffaella Semeraro, Giovanni Camastra, Viviana Langher, Mariella Pazzaglia, Luca Cacciotti

**Affiliations:** 1 Dipartimento di Psicologia Dinamica, Clinica e Salute, Università di Roma “Sapienza”, Via Degli Apuli 1, 00185 Roma, Italy; viviana.langher@uniroma1.it; 2 Dipartimento di Psicologia, Università di Roma “Sapienza”, Via dei Marsi 78, 00185 Roma, Italy; g.forte@uniroma1.it (G.F.); francesca.favieri@uniroma1.it (F.F.); francesca.agostini@uniroma1.it (F.A.); jasmine.giovannoli@uniroma1.it (J.G.); mariella.pazzaglia@uniroma1.it (M.P.); 3 Body and Action Lab, IRCCS Fondazione Santa Lucia, Via Ardeatina 306, 00179 Rome, Italy; 4 UOC di Cardiologia, Ospedale M.G. Vannini, 00189 Roma, Italy; luca.arcari88@gmail.com (L.A.); semerella.s@libero.it (R.S.); gcamastra@virgilio.it (G.C.); luca.cacciotti@figliesancamillo.it (L.C.); 5 Divisione di Cardiologia, Policlinico Casilino, Via Casilina, 00169 Roma, Italy; ilaria.passaseo@gmail.com

**Keywords:** acute myocardial infarction, life events, stress, Takotsubo cardiomyopathy, trigger

## Abstract

The onset of Takotsubo syndrome (TTS), also known as stress cardiomyopathy, is thought to be associated with some life events. This study focuses on clarifying life event characteristics and the role of triggers in the onset of TTS. Participants with TTS (*n* = 54) were compared to those with acute myocardial infarction (AMI; *n* = 52) and healthy individuals (*n* = 54). Using a modified version of the Interview for Recent Life Events, information about general life events perceived as stressful and triggers preceding the onset of a cardiac syndrome was collected. The assessment included the impact of these events as indicated by the participants and estimated by the interviewer; finally, the objective impact was considered. Although the number of events and the objective impact did not differ among the groups, patients with TTS reported a more negative perceived impact. Moreover, 61% of these patients objectively and subjectively reported a more stressful trigger before the onset of the disease (in the 24 h preceding the cardiac event) than those reported by patients with AMI. The dynamic between life events and individual responses could help differentiate TTS from other cardiovascular events, such as AMI. This study suggests that patients’ perception of some life events (whether triggers or general life events) could represent a possible marker of TTS.

## 1. Introduction

Takotsubo syndrome (TTS), also known as Takotsubo cardiomyopathy, broken-heart syndrome, or stress cardiomyopathy [1,2], is defined as a transient, reversible cardiac event that mimics an acute coronary syndrome, which involves the ventricular akinesis (i.e., apical, midventricular, basal or focal segments; [3]), without evidence of any artery obstruction or acute plaque rupture at angiographic detection [4,5]. Despite sharing the typical symptomatology of acute myocardial infarction (AMI), including chest pain, dyspnea, tachycardia, and syncope [1,6,7], coupled with the fact that different studies report similar short- and long-term outcomes [8,9,10], it seems to be characterized by fewer cardiovascular risk factors [11] and better prognosis [12] than AMI. 

The prevalence of TTS is 1–3% in all patients with an acute coronary syndrome manifestation [13,14,15,16,17]. Recent data have reported an annual TTS incidence between 50,000 and 100,000 cases in the general population [13]. These studies have also suggested that the prevalence increases by up to 10% in women [17,18], accounting for approximately 85–90% of patients with TTS. The age range for diagnosis is commonly between 67 and 70 years [8,16,17].

The definition of TTS as stress cardiomyopathy underlines the critical role of some life events as stressors in its onset [19]. Unlike other heart failure etiologies, such as AMI, a hallmark of TTS is the higher incidence of a trigger before the cardiac event, as reported in about 70% of patients [8,16,20,21]. The trigger occurs within 24 h before the onset of the cardiovascular event and could be emotional (e.g., grief, argument, separation) or physical (e.g., physical trauma, brain trauma, surgery). Despite the identification of many physical and emotional triggers (e.g., divorce, public speaking, conflict, severe fright, stress at work, physical illness) by case reports and cohort studies on TTS [11,22,23,24], no study has systematically assessed the nature and characteristics of triggers related to TTS onset [11,21].

Early studies suggested that both negative and positive life events could play a role in TTS onset [25,26]. These events are known to elicit significant emotional responses involving the sympathetic neurohormonal axis and parallel overstimulation of the catecholaminergic system by affecting the cardiovascular system [25,26,27]. Moreover, some authors have reported a high prevalence of TTS in patients with a history of psychopathological disorders (e.g., anxiety and depression) commonly associated with emotional dysregulation and a misperception of life and environmental events [8,28,29,30,31,32]. These findings potentially support the hypothesis that the nature of the triggers would not determine the onset of TTS per se, but rather that individual perception and response to trigger events could play a role in the onset of TTS [22,30,33]. Surprisingly, no study has analyzed how patients with TTS perceive the emotional impact of life events or triggers related to TTS onset. Accordingly, this study aims to understand the role and characteristics of both life events and triggers in TTS onset.

The main aim of this study was twofold. The first aim was to analyze life events and their impact on patients with a TTS diagnosis and compare their perception of these events to that of patients with an AMI diagnosis and individuals without a history of heart pathology. The second aim was to verify the differences in the presence and nature of triggers in patients with TTS and AMI.

We expected an overestimation of the emotional and stressful impact of life events in patients with TTS compared to those with AMI and healthy individuals. In the group comprising TTS patients, we also hypothesized a greater frequency of triggers, especially emotional ones, and a higher negative personal perception of their impact than in those with an AMI diagnosis.

## 2. Materials and Methods

### 2.1. Participants

One hundred and sixty people participated in the study. According to their health condition and diagnosis made by a cardiologist, the participants were divided into three groups:

(1) participants with TTS diagnosis (TTS; *n* = 54; Age: 71.5 ± 11.7; 3 M/51 F);
(2) participants with AMI diagnosis (AMI; *n* = 52 Age: 64.7 ± 9.5; 40 M/12 F);
(3) Healthy Participants (HP; *n* = 54 Age: 65.3 ± 12.1; 18 M/36 F).

### 2.2. Inclusion and Exclusion Criteria

For patients with TTS, the inclusion criteria were (1) acute onset of symptoms, (2) no culprit lesion on coronary angiography, (3) typical ‘apical ballooning’, (4) elevated cardiac biomarkers, and (5) normalized left ventricle systolic function on follow-up echocardiography. Diagnostic evaluation in TTS patients within our Institution has been previously described [12,34].

For patients with AMI, the inclusion criteria were (1) acute onset of symptoms, (2) elevated cardiac biomarkers, (3) a diagnosis of myocardial infarction made by a cardiologist, and consequent hospitalization.

For healthy participants, the inclusion criteria were (1) absence of any heart disease in clinical history, (2) an age similar to that of the two groups of patients.

To control possible confounding variables or aspects that could influence the dimensions assessed by the study, for all participants, general inclusion criteria were: (1) absence of severe chronic medical conditions (e.g., cancer, ictus, autoimmune diseases); (2) absence of neurological (e.g., epilepsy) and psychiatric diseases (e.g., schizophrenia, bipolar disorder, major depressive disorder) or diagnosis of dementia or other cognitive impairments.

To achieve the first aim of this study, life events and their impact were considered in the overall sample. To attain the second aim, only the two groups with hearth pathologies were considered to ascertain the trigger’s impact on patients with TTS and AMI.

### 2.3. Instruments

#### 2.3.1. Physiological Measures

In adherence to the European Guidelines for the assessment of blood pressure, systolic (SBP) and diastolic blood pressure (DBP), and heart rate (HR) were recorded through an electronic sphygmomanometer validated for self-measurement [35].

Moreover, the Body Mass Index (BMI; kg/m^2^) was calculated through participants’ weight and height measurements [36].

#### 2.3.2. Sociodemographic and Anamnestic Information

A face-to-face interview was conducted to collect sociodemographic (age, education, occupation, marital status) data. An investigation was also undertaken to seek information about medical history, including any psychiatric consultation or psychological treatment, as well as the presence of potential risk factors for cardiovascular pathologies. Specifically, the interview collected information about the medical conditions of hypertension, hypercholesterolemia, and hyperglycemia. Subsequently, information about lifestyles was reported: smoking habits (number of cigarettes smoked every day), alcohol consumption (number of glasses drunk daily), coffee consumption (number of cups drunk daily), and adequate physical activity (yes/no). All pharmacological treatments at the time of evaluation were also listed.

#### 2.3.3. Modified Version of Interview for Recent Life Events (IRLE)

The IRLE is a semistructured interview based on the Paykel Events Scale [37,38] wherein 63 life events are recorded in different categories: (1) work, (2) education, (3) economic problems, (4) health, (5) grief, (6) emigration, (7) family relationships, (8) social relationships, and (9) other events. The IRLE requires participants to analyze the life events, focusing on the six months preceding the interview or the disease’s onset. The time, frequency, and a succinct elucidation of each event are recorded. The interviewer-estimated the Independence and Objective Negative Impact of each reported event on two 5-points Likert scales. The Independence concerns the probability that the event may or may not have caused the illness. The Objective Negative Impact refers to the level of unpleasant impact, stress, or threat that the individual is expected to face due to the event, considering its nature and circumstances. While the participant’s characteristics must not influence it, the circumstances of its occurrence for both the patient and the event must be considered. However, this index expresses the limits of the interviewer’s characteristics. For these reasons, our group made some modifications to the IRLE. These modifications were aimed at better understanding the consequences of an event on individual health by also considering the subjective perception of the event (see Table 1). Three indices were analyzed in our adaptation of IRLE for each life event reported:

(a) The Indicated Impact by subject (Subject-Indicated Impact): the participant had to indicate how much he or she thinks the event adversely affected his or her life on a 5-points Likert scale (1: low negative impact; 5: high negative impact);
(b) The Objective Impact: this indicates the objective impact of the events on a 5-point Likert scale (1: low negative impact; 5: high negative impact). This index was obtained by administering the Paykel Events Scale and other life events suitable for Italian people (see Table 1) to a general sample of 512 Italian respondents (354 women and 158 males, mean age: 32.7 ± 13.56; mean years of education: 15.60 ± 3.33). This evaluation required the participants to respond to the following question: “how do you think this event generally affects the life of a person?”
(c) The Estimated Impact by the interviewer (Interviewer-estimated Impact): this indicates the negative impact that the event had on the participant life according to the interviewer’s view on a 5-point Likert scale (1: low negative impact; 5: high negative impact). This index deviates from the average of impacts estimated by two independent observers (inter-rater concordance: r = 0.976).

All three impacts were reported for each event listed by the participants.

The interview was aimed at investigating both the eventual triggers associated with the two cardiac events (TTS and AMI) and the main life events within the last six months reported by the participant. Considering the triggers, events characterized by intense negative emotions were classified as Emotional Triggers, whereas those entailing pain or high fatigue were classified as Physical Triggers. Considering the three impacts, two indices were considered: (1) the impact of the Trigger Events, (2) the mean impact of all the Life Events reported in the last six months.

### 2.4. Procedure

The research was conducted according to the Declaration of Helsinki principles, and was approved by the Ethics Committee of the Department of Dynamic and Clinical Psychology and Health studies of the University of Rome Sapienza (Prot. n. 0000664). A total of 97 patients were selected between January 2017 and January 2019 from the database at the Cardiology Department (54 TTS and 52 AMI patients), Vannini Hospital of Rome. A cardiologist selected patients who had been diagnosed and admitted to the Vannini hospital with TTS or AMI. All selected patients had no other chronic pathologies (cancer, diabetes, respiratory or neurological disorders). All had been discharged from the hospital for at least 3 months and were in good health. Patients diagnosed with TTS or AMI were briefly presented with the research and asked to participate in the evaluation voluntarily. Considering the number of patients in each group, we set the number of healthy controls as 54.

### 2.5. Data Analysis

Univariate Analyses of Variance (ANOVAs) were carried out to assess the differences between the three groups (HP, TTS, AMI) in age, years of education, some cardiac risk factors, e.g., BMI, cigarette consumption (n./day), alcohol consumption (glasses/day), caffeine consumption (cups/day), clinical recordings (SBP, DBP, HR), and Life Events’ Impact reported by the IRLE (Objective, Subject-Indicated, Interviewer-estimated). The planned comparisons were adopted to verify possible differences among groups.

An χ^2^ test was used to compare the differences in the percentage of the categorical variables among groups. Specifically, marital status, occupational status, presence of some cardiac risk factors (i.e., hyperglycemia, hypertension, hypercholesterolemia, family history of cardiovascular diseases), and nature of trigger (No trigger, Emotional Trigger, Physical Trigger) were tested. 

To analyze the characteristics of trigger events, ANOVAs for Trigger Impact (Objective, Subject-Indicated, Interviewer-estimated) were carried out between the two groups of patients (TTS, AMI).

Univariate Analyses of Covariance (ANCOVAs) were carried out on the indices of the impact of the Paykel interview, considering age and years of education as covariates to control the influence of these variables on the negative impacts of both life events and trigger events in the different groups. A Bonferroni’s correction was applied to reduce Type 1 error risk, and a *p* ≤ 0.02 was accepted.

## 3. Results

### 3.1. Demographics and Lifestyle Variables

Table 2 reported the differences between groups in demographic (age, year of education, gender, marital status, occupational status), cardiac risk factor (cigarettes, alcohol, and caffeine consumption, hyperglycemia, workout, hypercholesterolemia, hypertension, family history of CDVs, and Body Mass Index) and Clinical recordings (SBP, DBP, HR) of the three groups of participants (HP, TTS, AMI).

Participants showed significant differences in age (F2,157 = 5.90; *p* = 0.003) and years of education (F2,157 = 4.19; *p* = 0.02). The TTS group were older and had fewer years of education than both the AMI (Age: *p* = 0.003; Years of Education: *p* = 0.01) and HP groups (Age: *p* = 0.005; Years of Education: *p* = 0.02). The ANOVAs for the cardiac risk factors highlighted significant differences among the groups in BMI (F2,157 = 3.88; *p* = 0.02), smoking habits (F2,157 = 4.49; *p* = 0.01) and caffeine consumption (F2,157 = 7.34; *p* = 0.001), while no differences between groups emerged in alcohol consumption (F < 1; *p* = 0.47). The TTS group showed lower BMI than both the HP (*p* = 0.02) and AMI groups (*p* = 0.02). The TTS group had a lower cigarette consumption than the AMI group (*p* = 0.003). No differences between the AMI and HP groups emerged for both BMI (*p* = 0.95) and cigarette consumption (*p* = 0.11). The AMI group drank more cups of coffee per day than the TTS group (*p* = 0.0002); the coffee consumption was also marginally higher in the AMI group than the HP group (*p* = 0.04), while the TTS and HP groups were not significantly different (*p* = 0.07).

The ANOVAs for the clinical recordings showed significant differences between groups for SBP (F2,157 = 6.89; *p* = 0.001) and HR (F2,157 = 3.23; *p* = 0.04). No differences were highlighted for DBP (F2,157 = 1.60; *p* = 0.20). The AMI group showed lower SBP than both the HP (*p* = 0.04) and TTS groups (*p* = 0.0003), but the TTS and HP groups were not different (*p* = 0.11). Furthermore, the TTS group showed lower HR than the HP group (*p* = 0.02), while HR of the AMI group was not different from HR of both the TTS (*p* = 0.60) and HP groups (*p* = 0.06).

The χ^2^ for the cardiac risk factors did not show significant differences among the three groups of participants in the percentage of hyperglycemia and hypercholesterolemia or alcohol consumption and physical activity. The TTS group presented a lower CVD family history percentage than both the AMI and HP groups (*p* = 0.02). Both the groups with heart diseases reported a higher percentage of hypertension than HP (*p* = 0.0001).

### 3.2. IRLE’s Life Events’ Impacts

The ANOVA for the number of events reported by the groups in the IRLE showed only marginally significant differences (F2,157 = 3.44; *p* = 0.03). The TTS group indicated a higher number of events compared with the HP group (*p* = 0.01); while the TTS and AMI (*p* = 0.16), and AMI and HP groups (*p* = 0.25) did not indicate a different number of life events. 

**Figure 1:**
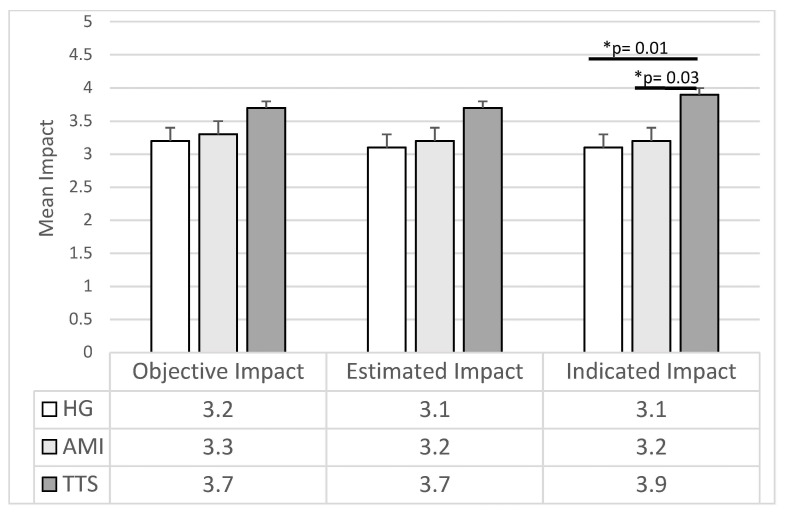
Mean and Std. Err. of the Impact of Life Events reported by the three groups of participants (*p* refers to ANCOVA results). HG: Healthy Group; AMI: Acute Myocardial Infarction; TTS: Takotsubo Syndrome.

The ANOVAs carried out on the impact of the Life Events highlighted a difference between groups in Interviewer-estimated Impact (F2,157 = 3.45; *p* = 0.03), and Subject I-dicated Impact (F2,157 = 4.65; *p* = 0.01). Specifically, the TTS group showed higher scores than both the HP (Interviewer-estimated Impact: *p* = 0.04; Subject-Indicated Impact: *p* = 0.01) and AMI (Interviewer-estimated Impact: *p* = 0.04; Subject-Indicated Impact: *p* = 0.01) groups. However, considering Bonferroni’s adjustment, these differences remain significant only for the Subject-indicated Impact. The AMI and HP groups did not differ (Interviewer-estimated Impact: *p* = 0.70; Subject-indicated Impact: *p* = 0.76). No differences among groups were observed in the Objective Impact (F2,157 = 2.49; *p* = 0.09) (see Figure 1, Table 3).

The ANCOVAs substantially confirmed the ANOVAs results. A significant difference in the Subject-indicated Impact (F2,157 = 3.95; *p* = 0.02) emerged, indicating a higher impact in the TTS group than both HP (F1,157 = 6.79; *p* = 0.01) and AMI (F1,157 = 5.15; *p* = 0.03) groups. The Interviewer-estimated Impact was disconfirmed (F2,157 = 2.56; *p* = 0.08).

### 3.3. IRLE’s Trigger Events’ Impact

Overall, 27% of patients with AMI (14 out of 52) and 61% of patients with TTS (33 out of 56) reported a trigger before the cardiac event. This difference was significant (χ^2^ = 4.93; *p* = 0.03). The TTS group reported 85% of these to be Emotional Triggers (28 out of 33) and 15% to be Physical Triggers (5 out of 33). The AMI group reported the same percentage of Emotional and Physical Triggers (50%, 7 out of 14). A significant difference between groups in the percentage of Emotional Triggers was observed (χ^2^ = 9.13; *p* = 0.003), while no significant differences emerged considering Physical Triggers (see Table 4).

**Figure 2:**
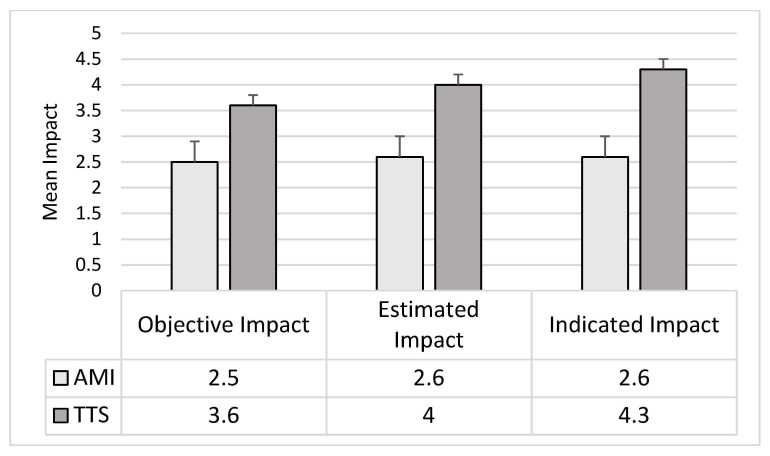
Mean and Std. Err. of the Trigger Impacts reported by the two groups of patients (*p* refers to ANCOVA results). AMI: Acute Myocardial Infarction; TTS: Takotsubo Syndrome.

The ANOVAs for the Trigger Event Impacts showed significant differences between the two groups in the levels of Objective Impact (F1,45 = 7.60; *p* = 0.01), Interviewer-estimated Impact (F1,45 = 11.78; *p* = 0.001; pη^2^ = 0.21), and Subject-indicated Impact (F1,45 = 17.79; *p* = 0.0001). The TTS group reported a higher score than the AMI group in all types of impact (Figure 2).

The ANCOVAs confirmed the ANOVAs results for all the impacts: Objective Impact (F1,43 = 5.81; *p* = 0.02), Interviewer-estimated Impact (F1,43 = 7.56; *p* = 0.01), and Subject-indicated Impact (F1,43 = 7.56; *p* = 0.01).

## 4. Discussion

Many studies have reported highly stressful life events as triggers of TTS [8,11,16,21,22,23,24], and in some cases, the IRLE [39] was adopted to analyze the impact of stressor patterns on the clinical manifestation of TTS [22]. Due to some structural limitations of this interview, it was impossible to explore all relevant aspects of the stressful events that may serve as triggers for TTS. In particular, the subjective perception of the event was not examined, which is surprising, given that each event can be considered a stressor according to subjective perception of it [40]. To overcome this shortcoming, we introduced a person’s subjective judgment parameter. Furthermore, we have partially attempted to remodel the objective weight of the events, estimating it on an Italian sample. Our adaptation of IRLE makes it possible to determine three critical aspects: (1) the supposed objective impact that a life event has on individuals, which determines the adaptive appraisal that the individual must adopt for an adequate response to that event [41]; (2) the subjective impact that a life event generates in each individual, which can be influenced by personal characteristics (e.g., personality traits, previous life experiences, personal resources, biological response [42,43,44]); and (3) the estimated impact of a life event as determined by the interviewer, using their knowledge of common adaptive responses by a reasonable individual.

The main results of this study showed that stressful events are perceived differently by patients with TTS when compared with both healthy people and patients with AMI. Patients with TTS tend to perceive life events more negatively than the interviewer. Surprisingly, the objective impact of life events was not greater in patients with TTS. Many studies have reported that chronic stress and multiple adverse life events are possible risk factors for TTS onset [24,29,32,45]. However, this is the first study to focus on the impact indicated by the subjects (i.e., participants’ perception of the events) compared to the objective impact of the event. Since patients with TTS do not report a higher number of stressful events than patients with AMI, this result may indicate that subjective perception rather than the presence of specific life events could signify a risk factor for TTS onset.

The higher SBP and HR values in patients with TTS in comparison with the other two groups of participants could be contingent on their emotional reactions [46], emotion dysregulation [47,48], and dysfunctional coping strategies generally associated with higher blood pressure levels [49].

Another aspect emerging from this study is that the modified version of the IRLE facilitates an improved definition of the trigger characteristics associated with cardiac events. Numerous studies have indicated that TTS is strongly associated with a stressor event, leading to a higher increase in catecholamine levels in TTS than in other heart diseases [27]. This increase could probably be attributed to sympathetic hyperactivation [27,50]. The relationship between a stressful trigger and the pathophysiological response of the cardiovascular system has prompted researchers to define a potential interaction between the brain and heart in response to stressful life events that cause TTS [51,52,53]. This brain–heart activation involves the limbic system (i.e., amygdala, insula, hippocampus, cingulate cortex), autonomic nervous system, and hypothalamic–pituitary–adrenal axis (HPA) [14,50,53].

Our results confirmed a higher frequency of triggers in patients with TTS compared with their counterparts with AMI [8,16,21], although the prevalence (61%) is lower than that reported by previous studies, which showed a prevalence rate between 70 [20] and 90% [54]. However, these differences are probably due to small sample sizes, as most previous studies comprised case reports (for a review [54]).

Emotional triggers occurred more frequently than physical triggers, which contrasts with the finding of a recent systematic review of 1330 case reports [54], in which physical triggers (e.g., drugs, surgery, brain trauma) were more common than emotional triggers in TTS. Emotional triggers (e.g., death of a close person, serious illness of a family member, dismissal, severe financial difficulties, etc.; see Table 1) could have a higher negative impact than physical triggers, thus explaining the high prevalence associated with TTS onset. Life events, which involve a relevant emotional response, would increase the activation of HPA as an automatic and adaptive response [55]. This activation could lead to cardiac syndromes by generating cascading events in vulnerable individuals [56].

Another interesting result is the higher impact of triggers in patients with TTS compared to those with AMI, shown by the IRLE in the three assessed indices. Although the patients reported a misperception of life events, characterized by a more negative perception, the trigger is objectively characterized by a higher stressful impact. According to this finding, possible markers of TTS onset could be the interaction between the person’s misinterpretation of life events and the objectively high impact of the trigger, generating physical distress. This result corroborates prior findings [28], suggesting an association between a change in emotional competencies and metacognitive strategy and the presence of emotional triggers in patients with TTS. Further studies should examine the role of metacognitive abilities and the impact or occurrence of emotional triggers in TTS onset, and the influence of these abilities on the subjective and estimated impact of life events.

These results should also be interpreted considering the general distinctive characteristics of patients with TTS and AMI. Unhealthy lifestyles, such as smoking habits and excessive body weight, were identified as risk factors for heart disease and are considered indicators of reduced life expectancy [57,58,59]. In our study, participants with TTS smoked fewer cigarettes per day and were less likely to smoke than those affected by AMI. Moreover, patients with TTS had a lower BMI than both healthy people and patients with AMI. These results confirm the findings of previous studies [11,26], which reported fewer cardiovascular risk factors in patients affected by TTS. Notably, the psychophysiological profile of patients with TTS differs from that of patients with AMI, thus indicating a different etiology of the two cardiac events.

Patients with TTS are expected to have healthier lifestyles than patients with AMI, but they presented an interpretation bias in life events. A similar number of stressful life events, characterized by comparable severity, affected people who developed TTS and AMI, but the negative perception of these events was higher in patients with TTS. This could be a typical trait in patients with TTS. In this case, it would be appropriate to investigate better psychological factors in patients with TTS, such as the strategies utilized for coping with stressful events and the ability to regulate their emotional responses.

Some limitations of this study should be considered when interpreting these results. The main limitation of this study is the gender imbalance between the two groups of patients, which renders its results preliminary. However, our data are consistent with those of epidemiological studies, which indicated a higher incidence of TTS diagnosis in women [60] and a higher incidence of AMI in men [61]. Further studies should consider balancing the groups for gender and age to facilitate interesting interpretations. For example, gender control could help analyze the role of some predisposing factors (e.g., lack of estrogen replacement or hormonal alteration) [62] in the relationship between stress and TTS. Moreover, age control could also elicit interesting outcomes regarding the effects of cardiac events in different stages of life. Moreover, an analysis of the psychological characteristics of patients affected by TTS could help specify aspects (e.g., coping strategies and emotional regulation) involved in stress management. This, in turn, could help explain why the onset of TTS is seemingly related to a poor response to adverse events.

Although an adequate number of patients were analyzed, the AMI group’s low number of triggers is another limitation that prevents additional inferences about trigger differences between patients with TTS and AMI.

Another possible limitation of this study is the absence of an analysis focused on positive events and their impact on individuals. Keeping in mind the studies that define TTS as a possible happy-heart syndrome [26,27], the analysis of positive events is an interesting research direction. The IRLE structure prevented the examination of this dimension; therefore, further studies are recommended.

Finally, the methodological limitations should be highlighted as there could be bias linked to the absence of a blinding strategy in the interviewers’ IRLE rating.

## 5. Conclusions

The present findings represent a first step in analyzing life events and their possible impact on patients with TTS. The impact of life events could be a psychological marker of this syndrome. The dynamic between life events and the individual stress response could influence an individual’s physiological activation and determine the onset of a cardiovascular event. Studies on this topic are critical for differentiating this cardiovascular syndrome from others. For this purpose, it is essential to better define the aspects involved in TTS onset. It would be interesting to strengthen this analysis, including the relationship between the brain and heart, for example, with an analysis of heart-rate variability, representing a gold standard measure for assessing autonomic activation. Furthermore, it would facilitate the definition of the relationship between cognitive and psychological characteristics and the physiological response of the individual [63,64].

## Figures and Tables

**Table 1:** Adaptation of the Paykel Events Scale (Paykel, 1971 [37]).

**(1) Miscarriage or stillbirth**	**(34) Child married against respondent’s wishes**
**(2) Natural calamities (e.g., earthquake, floods)**	(35) Marital separation not due to argument
**(3) Marital difficulties of a close family member**	(36) New person in household
**(4) Legal problems of a close family member**	(37) Retirement
**(5) Unemployment**	(38) Promotion
**(6) Occupational hazards (e.g., at work, etc.)**	(39) Change in work
**(7) Injuries (e.g., road accidents)**	(40) Cease steady dating (of at least three months)
(8) Scheduled abortion	(41) Move to another city
(9) Lawsuit	(42) Change in schools
(10) Child married with respondent’s approval	(43) Child leaves home (e.g., college)
(11) Death of spouse	(44) Menopause
(12) Jail sentence	(45) Minor legal violation
(13) Death of close family member (parent, sibling)	(46) Birth of live child (for mother)
(14) Spouse unfaithful	(47) Wife becomes pregnant
(15) Loss of personally valuable object	(48) Marriage
(16) Business failure	(49) Moving to the same city
(17) Divorce	(50) Birth of a child (father) or adoption
(18) Marital separation due to argument	(51) Begin education (full time or part-time)
(19) Court appearance for a serious legal violation	(52) Son drafted
(20) Unwanted pregnancy	(53) Arguments with boss or coworker
(21) Unemployed for one month	(54) Wanted pregnancy
(22) Death of a close friend	(55) To take an important exam
(23) Demotion	(56) Engagement of a son/daughter
(24) Begin an extramarital affair	(57) Become engaged
(25) Break engagement	(58) Move to another country
(26) Increased arguments with spouse	(59) Increased arguments with a resident family member
(27) Increased arguments with fiancé	(60) Change in work hours (much overtime, second job, much less than usual)
(28) Academic failure (important exam or course)	(61) Change in work conditions (new department, new boss, big reorganization)
(29) Marital reconciliation (after one partner left home)	(62) Argument with nonresident family member (in-laws, relatives)
(30) Hospitalization of a family member (serious illness)	(63) Minor personal physical illness (one that requires physician’s attention)
(31) Major financial difficulties (very heavy debts, bankrupt	(64) Take a large loan (more than one-half of a year’s earnings)
(32) Major personal physical illness (hospitalization or one month off work)	(65) Cease full-time education (graduate or drop out)
(33) Separation from a significant person (close friend or relative)	(66) Moderate financial difficulties (bothersome but not serious, i.e., increased expenses, trouble from bill collectors)

In **Bold** are the life events added by our group.

**Table 2:** Means (±SD) of sociodemographic and anamnestic characteristics of the three groups of participants.

	Healthy Participants	Acute Myocardial Infarction	Takotsubo Syndrome	χ^2^/F	*p*
*n*	54	52	54		
Age,	65.3 (12.1)	64.7 (9.5)	71.5 (11.7) ^a,b^	5.90	0.003
Years of Education	11.1 (5.1)	11.4 (4.6)	8.9 (5.1) ^a,b^	4.19	0.02
Female, *n* (%)	36 (66.7)	12 (23.1) ^a^	51 (94.4) ^a,b^	57.98	0.0001
**Marital Status, *n* (%)**				18.00	0.006
Married	36 (66.7)	39 (75.0)	35 (64.8)		
Single	3 (5.6)	4 (7.7)	1 (1.8)		
Divorced	5 (9.2)	7 (13.5)	1 (1.8)		
Widow	10 (18.5)	2 (3.8)	17 (31.6)		
**Occupational Status, *n* (%)**				30.50	0.0001
Employed	23 (42.6)	24 (46.1)	9 (16.7)		
Unemployed	6 (11.1)	3 (5.8)	15 (27.8)		
Retired	25 (46.3)	25 (48.1)	30 (55.5)		
**Cardiac Risk Factor**					
Smoking, *n* (%)	13 (24.1)	15 (28.8)	5 (9.2) ^a,b^	7.16	0.03
Smoking (n/die), mean (SD)	3.4 (6.5)	6.1 (12.6)	1.2 (4.1) ^a,b^	4.49	0.01
Diabetes, *n* (%)	5 (9.3)	4 (7.7)	3 (5.5)	<1	0.76
Hypercholesterolemia, *n* (%)	5 (9.3)	5 (9.6)	4 (7.4)	<1	0.91
Family History of CVD, *n* (%)	32 (59.3)	36 (69.2)	23 (42.6) ^a,b^	8.19	0.02
Hypertension, *n* (%)	25 (46.3)	43 (82.7) ^a^	45 (83.3) ^a^	23.26	0.0001
Body Mass Index, mean (SD)	26.1 (4.1)	26.1 (3.9)	24.1 (4.2) ^a,b^	3.88	0.02
**BP and HR, mean (SD)**					
SBP	137.9 (21.6)	128.9 (15.6) ^a^	144.8 (27.2) ^b^	6.8	0.001
DBP	77.4 (13.1)	73.5 (9.6)	75.4 (10.9)	1.63	0.19
HR	71.9 (12.0)	67.8 (11.0)	66.7 (9.9) ^a^	3.23	0.04

SD = standard deviation; CVD = cardiovascular disease; SBP = systolic blood pressure; DBP = diastolic blood pressure; HR = heart rate. χ^2^: ^a^ = differences from HP group (*p* < 0.05); ^b^ = differences from AMI group (*p* < 0.05).

**Table 3:** Statistical results of the Impact of Life Events indices reported by the groups.

	Healthy Participants	Acute Myocardial Infarction	Takotsubo Syndrome	F	*p*
Number of Events	2.2 (1.7)	2.6 (1.6)	3.0 (1.9)	3.44	0.03
Objective Impact	3.2 (1.6)	3.3 (1.3)	3.7 (0.9)	2.49	0.09
Subject-Indicated Impact	3.1 (1.7)	3.2 (1.4)	3.9 (0.9)	4.65	0.01
Interviewer-Estimated Impact	3.1 (1.6)	3.2 (1.3)	3.7 (1.0)	3.45	0.03

**Table 4:** Statistical results of the trigger indices reported by the groups.

	Acute Myocardial Infarction	Takotsubo Syndrome	χ^2^/F	*p*
**Trigger Events,** ***n* (%)**				
No Trigger	38 (73.0)	21 (38.9)	3.60	0.05
Emotional Trigger	7 (13.5)	28 (51.8)	9.13	0.003
Physical Trigger	7 (13.5)	5 (9.3)	<1	0.54
**Evaluation of Trigger Impact, mean (SD)**				
Objective Impact	2.5 (1.4)	3.6 (1.2)	7.60	0.01
Subject-indicated Impact	2.6 (1.5)	4.3 (1.1)	17.79	0.0001
Interviewer-estimated Impact	2.6 (1.5)	4.0 (1.2)	11.78	0.001
